# Trait rumination in post-stress growth among Chinese college students: the chain mediating effect of distress disclosure and perceived social support

**DOI:** 10.3389/fpubh.2023.1265405

**Published:** 2023-11-23

**Authors:** Zengjian Wang, Yining Xu, Huifang Zeng

**Affiliations:** ^1^Key Laboratory of Brain, Cognition and Education Sciences (South China Normal University), Ministry of Education, Guangzhou, China; ^2^School of Psychology, Center for Studies of Psychological Application, and Guangdong Key Laboratory of Mental Health and Cognitive Science, South China Normal University, Guangzhou, China

**Keywords:** trait rumination, distress disclosure, perceived social support, post-stress growth, chain mediating effect

## Abstract

**Background:**

Rumination has emerged as a significant factor contributing to personal growth following periods of stress or trauma. The present study aimed to investigate the relationship between trait rumination and post-stress growth (PSG) within the context of Chinese college students who encounter mild stressors in their daily lives. Moreover, we aim to evaluate the potential mediating roles played by both distress disclosure and perceived social support in this dynamic relationship.

**Method:**

All participants completed assessments using the Adolescent Self-Rating Life Events Checklist, Rumination Response Scale, Post-Stress Growth Inventory, Distress Disclosure Index, and Perceived Social Support Scale. Correlation and mediation analyses were conducted using SPSS PROCESS 4 MACRO.

**Results:**

All students reported experiencing mild psychological impacts as a result of negative life events in the past year and displayed moderate levels of PSG. There was no significant correlation observed between the effects of negative life events and PSG. Significant negative correlations were found between trait rumination and distress disclosure, perceived social support, and PSG. Distress disclosure and perceived social support jointly operated as sequential mediators in the relationship between trait rumination and PSG among all the participants. Qualitative analyses revealed different correlation patterns of high- versus low-ruminator.

**Conclusion:**

Trait rumination affects PSG both directly and indirectly, through its influence on distress disclosure and perceived social support. Our results emphasize the significance of actively participating in distress disclosure and nurturing a robust sense of social support to counteract the detrimental effects of rumination on post-stress growth among Chinese college students.

## Introduction

Individuals sometimes find themselves confronted with highly stressful and traumatic events (e.g., cancer, injury, pandemic), and although negative outcomes appear inevitable, the post-traumatic growth theory suggests that these experiences can also potentially lead to positive changes and psychological growth (1–3). However, for the majority of people, in general, distress typically emerges from low to mild-intensity stressors encountered in their daily lives. These stressors can also give rise to a phenomenon known as Post-stress growth (PSG), which bears similarities to post-traumatic growth in terms of the types of growth experienced when facing traumatic events (4–7).

Post-stress/traumatic growth is not an automatic outcome after experiencing stressful or traumatic events. Numerous factors, encompassing individual traits, the intricacies of challenging situations, emotional distress coping mechanisms, self-expression, the interplay of distant and immediate social-cultural influences, social support systems, and the extent of rumination, collectively contribute to shaping individuals’ perceptions of positive life changes (8–11). Rumination, recognized as a cognitive process exerting a direct impact, has consistently shown significant associations with post-traumatic growth across diverse studies conducted among individuals facing various stressful or traumatic events (11–16). However, the specific relation mechanism of trait rumination to PSG in the general college population is unclear, making this potential association crucial to understand.

### Trait rumination and post-stress growth

Rumination refers to the compulsive focus on the behaviors and thoughts that passively focus attention on one’s symptoms of distress and on all the possible causes and consequences of these symptoms (17). Traditionally, rumination has been associated with adverse outcomes, such as depression and anxiety (18). In recent years, most researchers have also uncovered a different perspective: the process of rumination, wherein individuals reflect on the impact and significance of a profoundly traumatic event, can lead to newfound wisdom and personal growth (19, 20). The majority of previous studies have demonstrated the influence of rumination on personal growth, with the nature of its effects being contingent upon factors such as the time elapsed since the events and the particular subcomponents of rumination (21–23). Notably, deliberate and reflective rumination that takes place relatively soon after exposure to trauma has been linked to a positive association with post-traumatic growth (24).

However, some studies have yielded inconsistent results regarding the connection between rumination and post-traumatic growth. To illustrate, Irie and their team identified a significant association between deliberate rumination and post-traumatic growth in parents of childhood cancer survivors; however, this connection did not manifest among parents of children with chronic diseases (25). In a study conducted by Shigemoto et al. (26), a negative, though non-significant, relationship emerged between brooding/reflective rumination and post-traumatic growth in college students who had experienced potentially traumatic events. Recently, Taku et al. (27) conducted a cross-country comparison of the connection between rumination and post-traumatic growth in 10 countries, revealing that deliberate rumination did not emerge as a robust predictor of post-traumatic growth in certain nations. Furthermore, Shigemoto (28) also found that there was a significant between-person variability in the effect of deliberate rumination on post-traumatic growth associated with the pandemic of coronavirus disease 2019 (COVID-19). These results may also indicate variations in rumination patterns under different circumstances and countries (25, 29).

Therefore, it is essential to acknowledge that the rumination observed in trauma survivors might manifest differently from that seen in individuals dealing with everyday stressful situations, potentially resulting in diverse effects on PSG. Furthermore, the use of the Ruminative Response Scale to assess self-reported trait rumination during daily life has demonstrated associations with uncertainty and a negative correlation with mental well-being (30). Accordingly, this study formulated the following hypothesis:

*Hypothesis 1: Trait rumination is negatively related to PSG.*

### The chain mediating effect of distress disclosure and perceived social support

According to the revised post-traumatic growth model, distress disclosure and social support were investigated as mediating variables in the relationship between cognitive engagement and the experience of post-traumatic growth (31). Distress disclosure entails the open sharing of negative thoughts and emotions with others (32). The psychological benefits of practicing distress disclosure include reduced stress levels and an enhanced sense of well-being and self-efficacy (33–36). The act of openly sharing one’s intrusive thoughts and emotions has been demonstrated to facilitate a shift towards deliberate and reflective rumination, thereby providing a chance to reassess life goals and create a meaningful narrative that fosters post-traumatic growth (3, 37).

The inclination toward rumination also influences distress disclosure. For example, Song et al. (38) discovered diverse correlation patterns among various subcomponents of rumination and distress disclosure in newly diagnosed gynecological cancer couples from China. Garrison et al. (39) found that daily-event rumination was positively related to daily-event disclosure among college students from the midwestern United States. However, they also observed a negative and non-significant correlation between brooding rumination and tendencies toward disclosure. Tsai et al. (40) uncovered that trait rumination played a moderating role in the effects of emotional disclosure and peer-helping writing on psychological distress among Chinese international students. Dong et al. (41) discovered that self-disclosure played a moderating role between intrusive rumination and post-traumatic growth among individuals within the first 3 months following an accidental injury in China.

Recent studies have pointed to distress disclosure as a robust predictor of post-traumatic growth, especially in the presence of strong social support (22, 42–44). Social support encompasses the assistance that individuals receive from various sources, including family, friends, and sometimes even strangers, during times of stress. Perceived social support, on the other hand, involves an individual’s expectations and evaluations of the availability of social support and their belief in the potential to receive such support (45, 46). Previous studies have confirmed that perceived social support significantly predicts PSG among Chinese junior high school students (47). Taku et al. (27) observed that irrespective of the characteristics of the samples, positive disclosure experiences were associated with posttraumatic growth in all 10 countries they examined.

Building on the established research supporting the mediator model of distress disclosure and perceived social support, and considering the sociocultural influence on distress disclosure (48), it remains crucial to empirically examine their combined impact on the relationship between trait rumination and PSG.

Therefore, this study proposes the following additional hypotheses:

*Hypothesis 2: Distress disclosure and perceived social support mediate the negative effect of rumination on PSG.*

**Figure 1:**
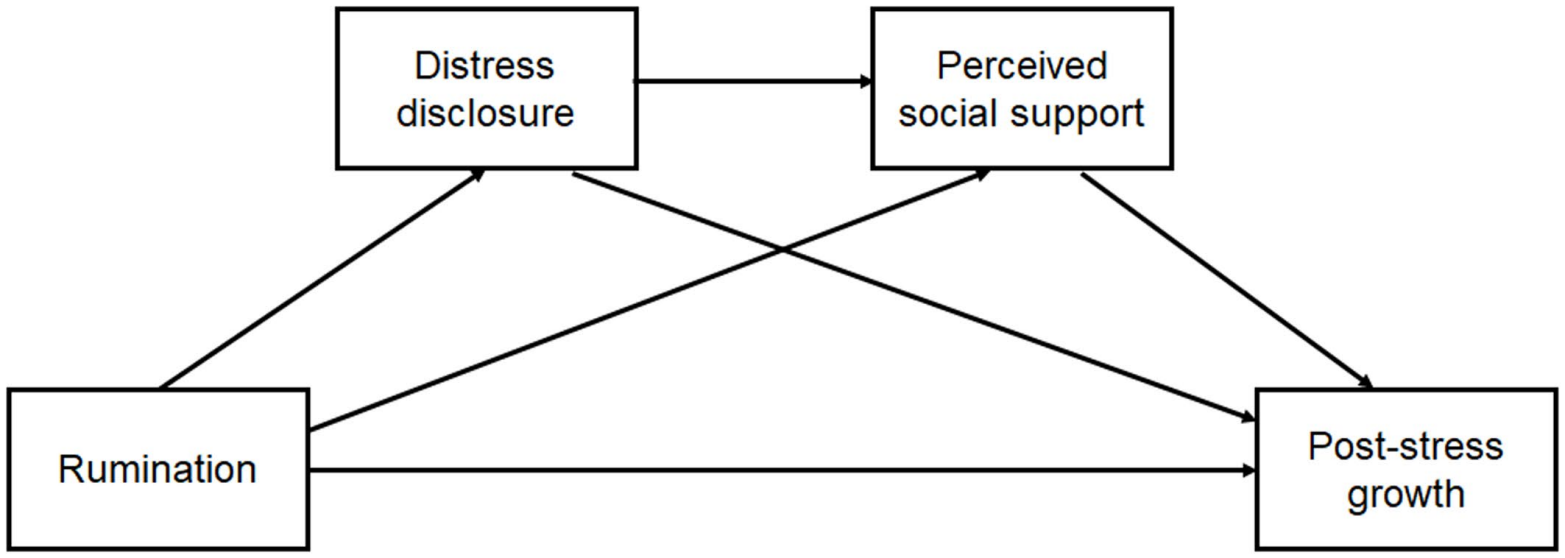
Hypothesized relationships between rumination, distress disclosure, perceived social support, and PSG.

Taken together, the current study first examined whether distress disclosure and perceived social support mediate the relation between trait rumination and PSG among Chinese college students (Figure 1).

## Materials and methods

### Participants

This study was approved by the local Ethics Committee of the School of Psychology, South China Normal University (SCNU-PSY-2022-274), and all participants provided informed consent. A total of 780 Chinese college students participated in the study from November 2022 to March 2023. After excluding invalid observations (e.g., missing data or errors), the final analysis included 629 participants (62.6% female). The sample primarily consisted of individuals aged 18–28 (50.9% were 18–21, and 40.7% were 22–24), with 50.5% coming from rural areas and 43.9% being only child.

### Research instruments

#### Adolescent self-rating life events checklist

To explore the occurrence of significant events, we employed the Adolescent Self-Rating Life Events Checklist (49) to evaluate the influence of adverse life events on Chinese college students over the preceding 12 months. This checklist comprises 27 items (e.g., “Relatives and friends suffer from acute or serious illness” and “Being discriminated against”) and encompasses six key dimensions: interpersonal relationship (1, 2, 4, 15, 25), learning pressure (3, 9, 16, 18, 22), punishment (17, 18, 19, 20, 21, 23, 24), loss (12, 13, 14), health adaptation (5, 8, 11, 27), and other (6, 7, 23, 24). Each item was assessed with a 5-point Likert scale to rate the influence of negative life events on Chinese college students: no influence (1), mild influence (2), moderate influence (3), severe influence (4), and extremely severe influence (5). The higher the total score, the greater the impact of negative life events. It is found that the scale is suitable for self-assessment in college students, with good reliability and validity, and the scale has been revised and tested many times (50–52). The internal consistency of this assessment tool was 0.97.

#### Ruminative responses scale

The Chinese Version (53) of the Ruminative Responses Scale (RRS) (54) was administered to assess how participants tend to respond to sad feelings and dysphoric symptoms. The RRS consisted of 22 items (e.g., “I go away by myself and think about why I feel this way” and “I think back to other times I have been depressed”) and included three dimensions: (1) brooding (5 items, item 1, 3, 6, 7, 8), (2) reflective pondering (5 items, 2, 4, 5, 9, 10), and (3) symptom rumination (12 items). Participants rated each item on a four-point scale ranging from 1 (never) to 4 (always), with higher mean scores indicating greater levels of trait rumination. This scale has been previously used in the Chinese population and has demonstrated strong reliability and validity (55–57). In the current study, the scale exhibited excellent reliability, with internal consistency coefficients of 0.951 for the total scale, 0.839 for brooding, and 0.848 for reflective pondering in the present sample.

#### Post-stress growth inventory

The Chinese Undergraduate’s Post-stress Growth Questionnaire (UPSGQ) was used to measure PSG (4, 58). The UPSGQ is comprised of 18 items, such as “I’ve learned to face problems with a positive attitude,” organized into four dimensions: Inter-personal relationship (5 items, e.g., items 1, 7, 10, 12, 17); Life view (5 items, e.g., items 4, 6, 9, 15, 18); Coping skills (5 items, e.g., items 3, 5, 8, 11, 13); Self-concept (3 items, e.g., items 2, 14, 16). Participants provided their responses on a 5-point Likert scale, where 1 indicated “Not at all” and 5 indicated “Exactly like me.” In the present sample, the internal consistency of the UPSGQ was found to be 0.898, indicating strong reliability.

#### Distress disclosure index

The Chinese version of Distress Disclosure Index (DDI) we used was revised by Zhen et al. (59) to measure comfort with self-disclosure (60, 61). The DDI comprises 12 items and is designed to gauge the extent to which an individual feels at ease discussing personally distressing information with others (62). A sample item is “I typically do not discuss things that upset me.” Respondents rate each item on a 5-point Likert-type scale, ranging from 1 (strongly disagree) to 5 (strongly agree). Kahn and Hessling (62) suggested that the 12 DDI items load on a single factor. A higher score on this scale indicates a greater willingness to disclose, implying effective emotional processing, and it has demonstrated robust psychometric properties (63). The internal reliability (Cronbach’s alpha) of the adapted version as reported by Wei et al. (60, 64) was 0.94; and in this study, it was 0.936.

#### Perceived social support scale

The Chinese version of the perceived social support scale (PSSS) was developed by Jiang (65); it has good reliability and has been used by most researchers (66–68). This scale assesses the perceived level of social support, with a higher total score indicating a greater sense of social support experienced by the individual. The PSSS covers three dimensions of support: family support (e.g., “My family can help me in concrete ways”), encompassing items 3, 4, 8, and 11; friend support (e.g., “My friends can really help me”), including items 6, 7, 9, and 12; and other support (e.g., “When I encounter problems, people will appear to support me”), which involves items 1, 2, 5, and 10. Participants rated their responses on a 7-point scale, with choices ranging from 1 (strongly disagree) to 7 (strongly agree). The Cronbach’s alpha coefficient for the PSSS was calculated to be 0.941, indicating a high level of internal consistency.

### Data analysis

The SPSS 22.0 software was used in the analysis. Tests of normality revealed that the study variables showed no significant deviation from normality [i.e., Skewness < |3.0| and Kurtosis < |10.0|; (69)]. To address the potential common method bias associated with self-administered questionnaires, this study utilized Harman’s single-factor test (70) and performed an exploratory factor analysis on all the items that covered the variables.

We first carried out descriptive statistics on demographic variables and five variables and then standardized the data of the five variables. To test the hypothesis, we used Pearson correlation analysis to explore the relationship between negative life events, rumination, PSG, distress disclosure, and perceived social support. Then, we used the SPSS PROCESS macro 4.1 software (71) to examine the mediating role of distress disclosure and perceived social support between rumination and PSG, which was specifically developed for testing the complex models. In PROCESS, model 6 software was applied for two mediators. Indirect effects were computed using a bias-corrected bootstrapping procedure. If the 95% confidence interval (CI) did not include 0, it meant that the mediation effect was significant. Gender, age, residence, and only child were included as covariates in the models.

To further explore the relationship between rumination and PSG, we conducted a quantitative analysis of our qualitative data. We divided participants into high- and low-rumination groups using the median-split method (RRS median = 47), as established by De Lissnyder et al. (72) and Koster et al. (73). Following this, we conducted independent sample t-tests to compare the levels of trait rumination between these two groups. To investigate the pathway linking rumination and PSG, we utilized Pearson correlation analysis to assess the association between trait rumination and its subcomponents with PSG within each respective group.

## Results

### Common method bias test

To examine common method biases and systematic errors due to self-rating questionnaires, the study used Harman’s single-factor test (70) and an exploratory factor analysis for all items containing five variables. The results showed that the first factor accounted for 22.46% of the total variation, i.e., lower than the threshold of 40% proposed by Podsakoff et al. (74). Although this result does not eliminate the possibility of common method variance, it suggests that common method bias is unlikely to confuse the interpretation of data analysis results.

### Descriptive analysis and correlations between overall variables

The Means, SDs, and Pearson correlations are presented in Table 1. The average total score for negative life events was calculated to be 71.30 ± 30.12 (range = 26–147). When considering the mean negative life events score after dividing the number of items, it equated to 2.74 ± 1.16. This result suggests a mild to moderate psychological impact experienced by Chinese college students in relation to these events.

**Table 1:** Descriptive statistics and correlation.

	M	SD	1	2	3	4	5	6
1. Rumination total	48.25	13.76	1					
2. Brooding rumination	11.98	3.51	0.90^**^	1				
3. Reflecting rumination	11.15	3.34	0.84^**^	0.74^**^	1			
4. Distress disclosure	41.70	9.91	−0.30^**^	−0.21^**^	−0.18^**^	1		
5. Perceived social support	63.06	12.78	−0.37^**^	−0.26^**^	−0.24^**^	0.64^**^	1	
6. PSG	71.31	9.13	−0.31^**^	−0.22^**^	−0.13^**^	0.43^**^	0.67^**^	1
7. Negative life events	71.30	30.12	0.02	0.04	0.04	−0.01	−0.02	−0.02

** *p* < 0.01.

The mean total scores for RRS were 48.25 ± 13.76 (range = 22–88), and the mean total scores for DDI were 41.70 ± 9.91 (range = 12–60), the mean total scores for PSSS were 63.06 ± 12.78 (range = 12–84), and the mean total scores for UPSGQ were 71.31 ± 9.13 (range = 23–90).

Table 1 shows that negative life events did not show significant correlations with any of the other variables. The total rumination score was significantly negatively correlated with distress disclosure (r = −0.30, *p* < 0.01), perceived social support (r = −0.37, p < 0.01), and PSG (r = −0.31, p < 0.01). The subcomponents of brooding rumination and reflective rumination exhibited similar relationship patterns with distress disclosure, perceived social support, and PSG. Distress disclosure was significantly positively correlated with perceived social support (r = 0.64, p < 0.01) and PSG (r = 0.43, p < 0.01). Perceived social support was significantly positively correlated with PSG (r = 0.67, p < 0.01).

### The chain mediating effects analyses

Rumination, distress disclosure, perceived social support, and PSG are significantly correlated, which meets the statistical requirements for further mediating effect analysis of rumination and PSG (75). After controlling for gender, age, residence, and only child, Model 6 in SPSS 22.0 compiled by Hayes (71) was used to analyze the mediating effect of distress disclosure and perceived social support in the relationship between rumination and PSG.

Table 2 shows the regression analysis results of the relationship between rumination and PSG, in which gender, age, residence, and only child are the control variables. The results show that rumination has a significant negative predictive effect on PSG (β = −0.38, *p* < 0.01). Hypothesis 1 has been tested. When distress disclosure and perceived social support are included in the regression equation, rumination significantly predicts distress disclosure (β = −0.29, *p* < 0.01) and perceived social support (β = −0.20, *p* < 0.01). Distress disclosure significantly predicts perceived social support (β = 0.58, *p* < 0.001). However, distress disclosure could not predict PSG (β = −0.01, *p* > 0.05). At this point, the direct effect value of rumination on PSG is significantly reduced (β = −0.08, *p* = 0.02). These results indicate that the chain mediating effect of distress disclosure → perceived social support is significant among the influences of rumination on PSG. Hypothesis 2 was confirmed.

**Table 2:** Regression analysis of the relationship between rumination and PSG.

Regress equation		Fitting index			Significance	
Result variable	Predictor variable	R	R2	F	β	t
PSG	Gender	0.34	0.11	15.72^**^	−0.09	−2.39^*^
	Age				0.02	0.49
	Residence				0.02	0.53
	Only child				−0.05	−1.28
	Rumination				−0.38	−8.10^**^
Distress disclosure	Gender	0.30	0.09	12.05^**^	0.01	0.22
	Age				0.01	0.31
	Residence				0.004	0.11
	Only child				−0.03	−0.72
	Rumination				−0.29	−7.56^**^
Perceived social support	Gender	0.67	0.45	85.67^**^	−0.05	−1.79
	Age				0.02	0.80
	Residence				0.001	0.03
	Only child				−0.04	−1.24
	Distress disclosure				0.58	18.66^**^
	Rumination				−0.20	−6.29^**^
PSG	Gender	0.68	0.46	74.95^**^	−0.06	−2.00^*^
	Age				−0.001	0.03
	Residence				0.02	0.60
	Only child				−0.02	−0.50
	Distress disclosure				−0.01	−0.25
	Perceived social support				0.64	16.09^**^
	Rumination				−0.08	−2.36^*^

* *p* < 0.05.

** *p* < 0.01.

**Figure 2:**
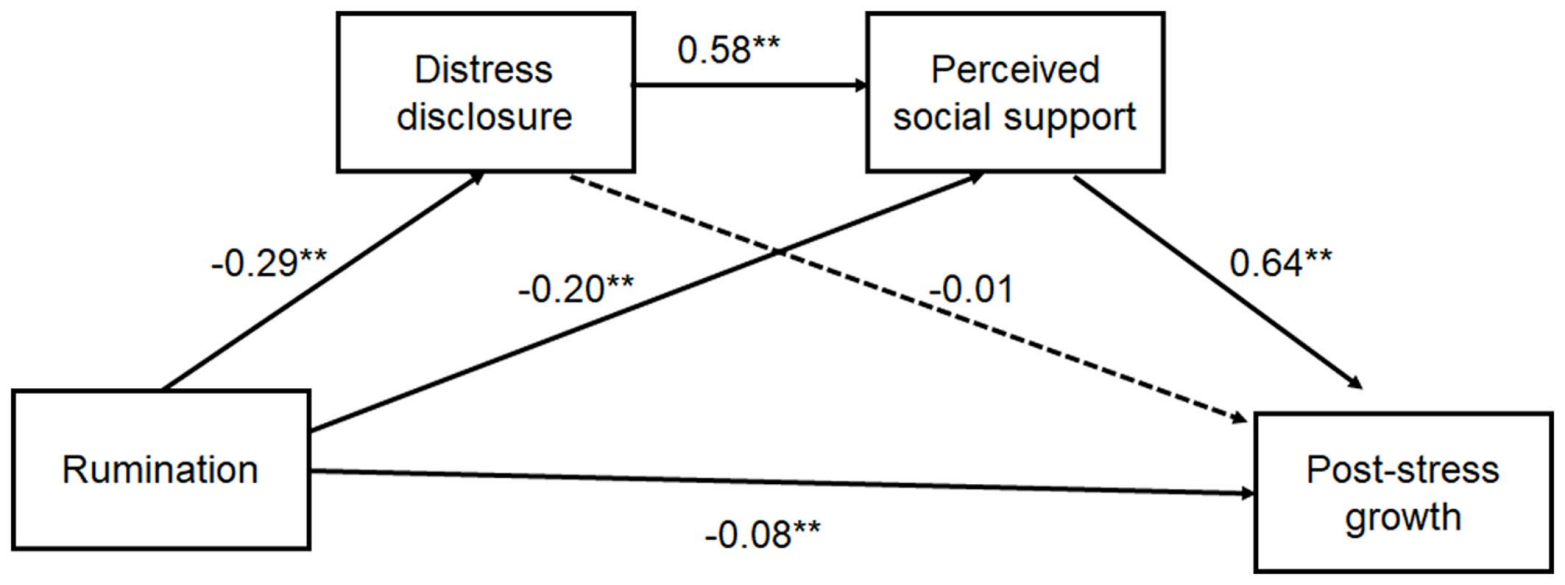
The chain mediating effect of distress disclosure and perceived social support.

Table 3 shows the chain mediating effect value of distress disclosure and perceived social support in the relationship between rumination and PSG. Figure 1 is a chain mediating model between rumination and PSG. Table 3 and Figure 2 show that distress disclosure and perceived social support play a significant chain mediating role between rumination and PSG. The total effect value of rumination on PSG is −0.255, the direct effect value of rumination on PSG is −0.087 and the total standardized mediating effect value is −0.168. The ratio of the total standardized mediating effect to the total effect is 65.88%. The mediating effect is composed of three indirect effects: path 1: rumination → distress disclosure → PSG (0.01), path 2: rumination → perceived social support → PSG (−0.07), path 3: rumination → distress disclosure → perceived social support → PSG (−0.11). The ratios of the three indirect effects to the total effect are 4.31, 27.45, and 42.35% for paths 1, 2, and 3, respectively. The two indirect effects of path 2 and path 3 reach a significant level because the 95% confidence interval of the above indirect effects does not contain the zero value. However, path 1 was not significant as the 95% confidence interval of the above indirect effects contains the zero value. Comparison 3 shows that the bootstrap 95% confidence interval for the difference between indirect effects 2 and 3 contains a 0 value, indicating that there is no significant difference between them. Using the same method to compare 1 and 2, the bootstrap 95% confidence interval for the difference does not contain a 0 value, which means path 1 is significantly different from paths 2 and 3.

**Table 3:** Distress disclosure and perceived social support in the mediation effect analysis.

	Indirect effects	Boot SE	Boot LLCI	Boot ULCI	Relative mediation effect
Total indirect effect	−0.168	0.03	−0.22	−0.12	65.88%
indirect effect 1	0.01	0.01	−0.01	0.04	4.31%
indirect effect 2	−0.07	0.02	−0.11	−0.03	27.45%
indirect effect 3	−0.11	0.02	−0.15	−0.07	42.35%
Compare 1	0.082	0.026	0.032	0.132	
Compare 2	0.119	0.026	0.072	0.172	
Compare 3	0.038	0.026	−0.012	0.090	

All variables in the model have been standardized. Boot SE, Boot LLCI, and Boot ULCI refer, respectively, to the standard error and the lower and upper limits of the 95% confidence interval of the indirect effects estimated by the percentile bootstrap method with deviation correction. Indirect effect 1: rumination → distress disclosure → PSG; indirect effect 2: rumination → perceived social support → PSG; indirect effect 3: rumination → distress disclosure → perceived social support → PSG. Compare 1: indirect effect 1–indirect effect 2; compare 2: indirect effect 1–indirect effect 3; compare 3: indirect effect 2–indirect effect 3.

The following results indicate that rumination could not predict PSG through the single mediating effect of distress disclosure but could through the single mediating effect of perceived social support as well as through the chain mediating effect of distress disclosure and perceived social support.

### Quantitative analysis of qualitative data

In contrast to the low rumination group, the high rumination group exhibited higher levels of total trait rumination [t_(627)_ = −35.66, *p* < 0.001], brooding rumination [t_(627)_ = −28.40, *p* < 0.001], and reflective rumination [t_(627)_ = −24.40, *p* < 0.001], along with reduced levels of distress disclosure [t_(627)_ = 6.00, *p* < 0.001], perceived social support [t_(627)_ = 8.00, *p* < 0.001], and PSG [t_(627)_ = 7.07, *p* < 0.001]. Furthermore, Pearson correlation analysis revealed that only reflective rumination demonstrated a significantly positive relationship with PSG within the high rumination group (r = 0.14, *p* = 0.02). A comprehensive summary of these results is presented in Table 4.

**Table 4:** Variations and correlations in variables between high and low ruminators.

		1	2	3	4	5	6
High ruminators (*N* = 306)	1. RRS total	1					
	2. Brooding	0.74^**^	1				
	3. Reflective	0.70^**^	0.44^*^	1			
	4. UPSGQ	−0.12^*^	−0.03	0.14^*^	1		
	5. DDI	−0.25^**^	−0.12^*^	−0.09	0.44^**^	1	
	6. PSSS	−0.25^**^	−0.07	−0.05	0.66^**^	0.64^**^	1
	Mean ± SD	59.81 ± 8.78	14.69 ± 2.37	13.54 ± 2.53	68.76 ± 10.07	39.32 ± 10.37	59.04 ± 13.94
Low ruminators (*N* = 323)	1. RRS total	1					
	2. Brooding	0.76^**^	1				
	3. Reflective	0.68^**^	0.47^**^	1			
	4. UPSGQ	−0.23^**^	−0.03	<0.001	1		
	5. DDI	−0.11	0.02	0.06	0.30^**^	1	
	6. PSSS	−0.19^**^	−0.002	−0.01	0.57^**^	0.56^**^	1
	Mean ± SD	37.30 ± 7.00	9.42 ± 2.28	8.88 ± 2.26	73.72 ± 7.38	43.96 ± 8.90	66.87 ± 10.22

RRS, ruminations response scale; Brooding, brooding rumination; reflective, reflective rumination; UPSGQ, Undergraduate’s PSG Questionnaire; DDI, distress disclosure index; PSSS, perceived social support scale.

** *p* < 0.01.

* *p* < 0.05.

## Discussion

Previous research has examined the relationships between rumination and post-traumatic growth; however, there has been limited investigation into the association between rumination and PSG. Additionally, a substantial number of these studies have centered on clinical patients or individuals who have encountered trauma. Consequently, it remains uncertain whether these findings can be extended to individuals experiencing low or mild stressors. In this study, our findings revealed a significant adverse association between trait rumination and PSG in the context of Chinese college students. Additionally, we identified distress disclosure and perceived social support as intermediary variables that play a role in mediating the connection between trait rumination and PSG. These findings hold significant implications for institutions seeking to foster PSG among Chinese college students who experience mild to moderate stress as part of their everyday experiences.

### The negative association between trait rumination and PSG

In accordance with Hypothesis 1, we made a noteworthy and significant finding: there were substantial negative correlations observed between trait rumination, which encompasses both its subcomponents of brooding rumination and reflecting rumination, and PSG across all participants.

One plausible explanation for the detrimental effect of trait rumination on PSG observed in our study may be attributed to the choice of data collection instrument. In our research, we specifically utilized the Rumination Response Style questionnaire (17). This assessment tool incorporates subcomponents of brooding and reflection and was intentionally designed to gauge the intensity of depressive rumination (76). In contrast, some previous investigations used the Event-Related Rumination Inventory (77), which encompasses both intrusive and deliberate rumination and is recognized as a measure of posttraumatic rumination (24). Brooding and reflection are conceptualized as trait-like thinking styles or personality characteristics (78), whereas intrusive and deliberate rumination are specific to a particular posttraumatic event (64, 79–85). In the limited studies where these rumination types were examined concurrently, deliberate rumination emerged as the sole unique positive predictor, while brooding was identified as the sole unique negative predictor of posttraumatic growth (12, 13, 86).

Another possible explanation relates to the participants recruited for our study. Specifically, all individuals in our research had experienced mild negative life events within the past 12 months. However, prior research has suggested that individuals tend to shift from automatic rumination to more deliberate forms of cognitive processing predominantly after experiencing significant stressful or traumatic events (87). After dividing all the participants into high-ruminators and low-ruminators, we only found a significant positive relationship between reflective rumination and PSG among high ruminators. Reflective rumination denotes a conscious, introspective process wherein an individual deliberately directs their thoughts inward, engaging in adaptive problem-solving. It serves as a relatively harmless form of rumination, which is a fundamental process essential for the emergence of PSG (87). Recruiting a sample of individuals who have experienced highly stressful or traumatic events to further investigate the relationship is an important direction for future research. Additionally, it remains critical to explore strategies aimed at mitigating brooding tendencies and fostering reflective rumination among college students, such as mindfulness interventions, given their substantial influence on personal growth (18).

### The mediating effect of distress disclosure and perceived social support

We further found that the relationship between trait rumination and PSG was mediated by distress disclosure and perceived social support. Consistent with the posttraumatic growth model (88), we found a significant positive correlation between distress disclosure and PSG. Individuals who engaged in a higher degree of disclosure tended to become more emotionally expressive, a crucial aspect of personal growth (82). However, distress disclosure, by itself, could not serve as an independent indirect mediator in the link between trait rumination and PSG. The result may be attributed to the negative correlation between trait rumination and distress disclosure, as individuals with high levels of rumination showed lower levels of distress disclosure, perceived social support, and PSG. We postulate that the negative correlation may be due to the limited acceptance of verbalizing distress as a method of emotional regulation within Asian cultures, which place a strong emphasis on emotional control (48). Previous studies found that some pressure to disclose may facilitate PTG but that too much pressure to disclose may impede growth (89). PSG is more likely to occur with positive distress disclosure, where the individual perceives a strong connection with their social network and feels relief and support following their discussion of the event (27). Future studies should explore the potential obstacles that individuals with a higher propensity for rumination may encounter when attempting to convey their distress to others within the context of Chinese cultures.

Consistent with a previous study (47), we also found that perceived social support, independently, could serve as an indirect mediator in the relationship between trait rumination and PSG. Perceived social support has the potential to mitigate the adverse effects of rumination on individuals, enhancing positive experiences and fostering personal growth (23). Social support creates a secure space for individuals to openly discuss stressful experiences, their associated perceptions, and emotions. It enables them to reframe traumatic events and reconstruct their worldviews, ultimately fostering their growth in the aftermath of stress or trauma (1, 90). Perceived social support has been found to have a more significant impact on growth than actual social support, underscoring its importance in the process (47, 91, 92). Mental health professionals in college settings can develop interventions targeted at enhancing the perception of social support to enhance the mental well-being of college students who are currently experiencing moderate stress due to negative life events (6, 93, 94).

### Negative life events and PSG

In our study, we did not observe any correlation between the effects of negative life events and the other variables, particularly with respect to PSG. In fact, the relationship concerning negative life events and posttraumatic growth was not entirely inconsistent (95). Jayawickreme et al. (96) discovered that experiencing negative life events predicted increases in state posttraumatic growth but not overall posttraumatic growth over 1 year. Fraus et al. (95) found that the total number of traumatic events was not significantly associated with posttraumatic growth. The Posttraumatic Growth model (3) also indicates that PTG does not always align with stress but rather emerges during the experience of psychological struggle when facing challenged worldviews. Future studies should underscore the significance of investigating processes related to PSG using suitable research designs, analytical strategies, and timeframes (96).

### Limitations and future directions

This study provides theoretical guidance for Chinese college students in managing stress and promoting PSG. However, several limitations warrant attention. Firstly, the measurement of rumination and distress disclosure was relatively simplistic and reliant on self-reporting, which may not fully capture the intricate nature and significance of these factors in relation to PSG among Chinese college students. To enhance the robustness of the findings, future studies are encouraged to explore diverse measurement approaches. Secondly, the study’s sample size is relatively limited, and its findings may not be generalizable to the broader population of college students. To increase the generalizability of the results, future studies should consider expanding the sample size to yield more convincing outcomes. Thirdly, this research is constrained by objective factors and employs a cross-sectional study design. While previous research forms a strong foundation for this study, it does not completely elucidate the mechanisms through which trait rumination and distress disclosure influence PSG. In future investigations, a longitudinal research design could be employed to explore the causal relationships between these variables.

## Conclusion

In conclusion, by addressing these limitations and incorporating more robust research methods, future studies can offer a more comprehensive understanding of how rumination, distress disclosure, and perceived social support impact the PSG of Chinese college students. This, in turn, could significantly contribute to the formulation of more effective strategies for stress management and personal growth within this population.

## Data availability statement

The original contributions presented in the study are included in the article, further inquiries can be directed to the corresponding author.

## Ethics statement

The studies involving humans were approved by the local Ethics Committee of the School of Psychology, South China Normal University (SCNU-PSY-2022-274). The studies were conducted in accordance with the local legislation and institutional requirements. The participants provided their written informed consent to participate in this study.

## Author contributions

ZW: Conceptualization, Formal analysis, Funding acquisition, Writing – original draft, Methodology, Supervision. YX: Conceptualization, Data curation, Formal analysis, Writing – original draft. HZ: Formal analysis, Writing – original draft.
